# Astaxanthin prevents ischemia-reperfusion injury of the steatotic liver in mice

**DOI:** 10.1371/journal.pone.0187810

**Published:** 2017-11-09

**Authors:** Shaowei Li, Terumi Takahara, Masayuki Fujino, Yasuyuki Fukuhara, Toshiro Sugiyama, Xiao-Kang Li, Shiro Takahara

**Affiliations:** 1 Division of Transplantation Immunology, National Research Institute for Child Health and Development, Tokyo, Japan; 2 Department of Advanced Technology for Transplantation, Osaka University Graduate School of Medicine, Osaka, Japan; 3 Research Center of Molecular Biology, School of Basic Medical Sciences, Inner Mongolia Medical University, Hohhot, Inner Mongolia, China; 4 Clinical Medicine Research Center of Affiliated Hospital, Inner Mongolia Medical University, Hohhot, Inner Mongolia, China; 5 Third Department of Internal Medicine, University of Toyama, Toyama, Japan; 6 AIDS Research Center, National Institute of Infectious Diseases, Tokyo, Japan; 7 Division of Medical Genetics, National Center for Child Health and Development, Tokyo, Japan; National Institutes of Health, UNITED STATES

## Abstract

Steatosis has a low tolerance against ischemia-reperfusion injury (IRI). To prevent IRI in the steatotic liver, we attempted to elucidate the protective effect of astaxanthin (ASTX) in the steatotic liver model by giving mice a methionine and choline-deficient high fat (MCDHF) diet. Levels of lipid peroxidation and apoptosis, the expression of inflammatory cytokines and heme oxygenase (HO)-1, in the liver were assessed. Reactive oxygen species (ROS), inflammatory cytokines, apoptosis-related proteins and members of the signaling pathway were also examined in isolated Kupffer cells and/or hepatocytes from the steatotic liver. ASTX decreased serum ALT and AST levels, the amount of TUNEL, F4/80, or 4HNE-positive cells and the mRNA levels of inflammatory cytokines in MCDHF mice by IRI. Moreover, HO-1 and HIF-1α, phosphorylation of Akt and mTOR expressions were increased by ASTX. The inflammatory cytokines produced by Kupffer, which were subjected to hypoxia and reoxygenation (HR), were inhibited by ASTX. Expressions of Bcl-2, HO-1 and Nrf2 in hepatocytes by HR were increased, whereas Caspases activation, Bax and phosphorylation of ERK, MAPK, and JNK were suppressed by ASTX. Pretreatment with ASTX has a protective effect and is a safe therapeutic treatment for IRI, including for liver transplantation of the steatotic liver.

## Introduction

Due to the increasing prevalence of non-alcoholic steatotic liver disease, a greater proportion of donor livers have steatosis becomes up to more than 30%, which is attributed to poor outcomes such as primary graft non-function and dysfunction. Moreover, a donor liver with steatosis of more than 60% is hopelessly unsuitable for liver transplant [1]. However, due to the extremely limited availability of organs for liver transplant and long waiting lists for patients who need transplantation, the use of extended criteria donor (ECD) liver allografts has become more common [2]. The risk of graft failure and dysfunction after transplantation when using steatotic ECD livers has led to an increase in ischemia-reperfusion injury (IRI) [3]. Although there is still some way to go, efforts are continuing to find efficient methods to reduce IRI in the steatotic liver in order to improve clinical outcomes of steatotic liver grafts [4]. The development of protective strategies through optimizing preconditioning to reduce the negative effects of IRI in steatotic livers is urgently needed.

Various factors contribute to injury associated with inflammation in hepatic IRI, including Kupffer cell activation, up-regulation of pro-inflammatory cytokines, chemokines, adhesion molecules, oxidative stress and so on [5, 6]. In addition, it has been reported that steatotic livers are less tolerant when subjected to IRI, because the lipid accumulation in the steatotic liver produces a much more reactive oxygen species (ROS) resulting in mitochondrial dysfunction [7] and subsequently leads to hepatocyte apoptosis or necrosis [8]. Ischemia stimulates the activation of Kupffer cells, which are the principal sources of ROS formation during the subsequent period of reperfusion [9]. Oxidant stress induced by Kupffer cells is a critical factor in hepatocyte apoptosis in reperfusion [10]. Some research has shown that rat livers subjected to damage by IRI experienced massive necrosis that may be induced by tumor necrosis factor (TNF)-α-regulated cell death [11, 12].

Astaxanthin (ASTX) is a xanthophyll carotenoid, which can be produced by a variety of seaweed and microorganisms [13]. ASTX has many antioxidant properties because it has a unique and remarkable molecular structure of hydroxyl and keto moieties on each ionone ring [14]. Antioxidant properties of ASTX have been previously observed in the plasma, eye and liver of rats fed with microalgal biomass, which contained ASTX, dispersed in olive oil [15]. Further, levels of antioxidant enzymes were up-regulated when ASTX was administered in ethanol-induced gastric ulcer rats [16]. Furthermore, an *in vitro* study has shown the beneficial effect of ASTX in tubular epithelial cells stimulated by high glucose, which induced inflammation and oxidative stress [17].

In this study, we tested the effect of ASTX on acute liver IRI in a steatotic mouse liver model. We then investigated the anti-inflammatory effect of ASTX in Kupffer cells and the anti-apoptotic effect in hepatocytes with hypoxia and reoxygenation (HR).

## Materials and methods

### Ethics statement

All mice were housed in individual cages within sound-attenuated, temperature-controlled isolation chambers on a 12:12 light-dark cycle (lights on at 7 am) at 25°C ambient temperature (except where stated otherwise). Mice used for survival studies were examined daily by animal care takers. To support strengthening of weak mice, daily changed paste, of normal food pellets, was provided in addition to pellets. Mice were treated in accordance with the Japanese Laws and the Guidelines for Humane Endpoints for Animals Used in Biomedical Research, National Research Institute for Child Health and Development and all protocols were previously subjected and approved by the Ethical Committee of the National Research Institute for Child Health and Development. Mice were euthanized if observed in a moribund state as defined by hypoactivity, sudden weight loss, decreased body temperature, or respiratory distress. Moribund mice were euthanized by CO2 inhalation.

### Preparation of ASTX

ASTX was a gift from AstaReal Co., Ltd. (Tokyo, Japan), and was dissolved in olive oil to obtain suspension and stored at 4°C in a dark place.

### ASTX administration

The mice were administrated p.o. with olive oil or ASTX (25mg/kg) three times for 48 hours, 24 hours, and 40 minutes before ischemia and reperfusion. Kupffer cells and steatotic hepatocytes were pretreated *in vitro* with 10μM ASTX dissolved in dimethyl sulfoxide for 24 hours.

### Animals grouping and experimental protocol

Eight weeks old male C57BL/6 mice, purchased from Shizuoka Laboratory Animal Center (Shizuoka, Japan) were raised in automatically controlled light and temperature feeding room. To establish the steatotic liver mice model, mice were fed with the diet which methionine, choline-deficient and high fat (MCDHF) for 21days. To be the specific, MCDHF contains 25% of oil, 20% of sugar and 55% of MCD. We chose 15 minutes ischemia and 3 hours reperfusion to performer animal ischemia/reperfusion experiments. Steatotic liver mice were randomly divided into 3 groups and underwent:

Group 1, MCDHF group: mice received olive oil by gavage with shame operation;
Group 2, MCDHF IR group: mice received olive oil by gavage before IR;
Group 3, MCDHF IR + ASTX group: mice received ASTX (25 mg/kg) for 48, 24hrs and 40mins by gavage before IR. The mice were sacrificed after 15mins ischemia and 3hrs reperfusion.

### Establish model of hepatic ischemia reperfusion in steatotic liver

The steatotic liver model of hepatic IR was established according to our previous work [18]. Specifically, the steatotic liver mice ischemia was subjected by occluding the hepatic portal vein with a microvascular clamp for 15mins followed by 3hrs reperfusion. After reperfusion close the abdomen incision immediately. The mice were sacrificed after 15mins ischemia and 3hrs reperfusion, the specimen of liver tissues and sera were harvested. All animal experiments were performed according to the recommendations of the Committee on the Care and Use of Laboratory Animals at the National Research Institute for Child Health and Development. The protocol was approved by the Committee on the Care and Use of Laboratory Animals at the National Research Institute for Child Health and Development (Permit Number: 2002–003). All surgery was performed under ether anesthesia, and all efforts were made to minimize suffering.

### Histopathological examination

Liver tissue was obtained for histopathological examination. In detail, specimen of removed liver tissue fixed by 10% paraformaldehyde, paraffin embedded and sliced into 4μm pieces. The severity of necrosis in steatotic liver was measured by hemotoxylin-eosin (HE) staining and quantified by WinRoof V6.1 software (Mitani Corporation, Tokyo, Japan).

### Immunohistochemical examination of tissue sections

Immunohistochemistry was performed to paraffin sections by using monoclonal antibody against F4/80 (Abcam, Cambridge, MA) and polyclonal antibody against 4-HNE (Abcam, Cambridge, MA). After Immunohistochemical staining, specimens were captured under microscope with a camera (Olympus, Tokyo, Japan).

### TUNEL assay

In order to measure hepatocyte apoptosis, the TUNEL assay was performed upon paraffin sections to label the 3’-end of fragmented DNA by apoptosis detection kit according to the manufacturer’s instructions (CHEMICON, Billerica, MA).

### The serum of ALT, AST enzyme-activity

Whole blood samples were harvested by storing for 40min at room temperature after 15min ischemia and 3hr reperfusion. Sera were collected by centrifuged at 1800g for 20min at 4°C. The serum ALT and AST were measured by an automatic biochemical analyzer (DRI-CHEM 3500i, FUJIFILM, Japan) according to the manufacturer’s protocol.

### RNA preparation and qRT-PCR analysis

RNA was extracted from approximately 100 mg frozen tissues by using RNeasy Mini Kit (QIAGEN, Valencia, CA). 800ng RNA of each sample was reverse transcribed to cDNA by using Prime Script RT reagent Kit (RR037A, TAKARA, Japan). Quantitative RT-PCR was performed by primer/probe system (the sequences are listed in Table 1) using a Applied Biosystem PRISM7900 (ABI Japan, Co., Ltd., Tokyo, Japan). The thermal cycling conditions of Realtime-PCR cycles were as follows, 2 min at 50°C, 15 min at 95°C and 40 cycles of 30 s at 95°C, 1 min at 60°C, and 2 min at 25°C. The 2^-ΔΔCt^ method was used to quantify the relative expression, and normalized the results by subtracting the 18S expression.

**Table 1:** Primer sequences and probes used in this study.

Genes	Forward (5^’^–3^’^)	Reverse (5^’^–3^’^)	Probe
IL-6	CTGCAAGTGCATCATCGTTGT	TGTCTATACCAC TTCACAAGTCGGA	CAGAATTGCCATTGCACAACTCTTTTCTCA
TNF-α	TGTCTACTGAACTTCGGGTGAT	AACTGATGAGAGGGAGGCCAT	TCCCCAAAGGGATGAGAAGTTCCCAA
iNOS	CAGTGGAGAGATTTTGCATGACA	CCCCAAGCAAGACTTGGACTT	CCACAAGGCCACATCGGATTTCACTT
Nrf2	TGATGGACTTGGAGTTGCCA	CCAAGATCTATGTCTTGCCTCCA	CAGTCCCAGCAGGACATGGATTTGATTG
OPN	CCCGGTGAAAGTGACTGATTCT	GATTCTGCTTCTGAGATGGGTCA	AGCTCAGAGGAGAAGCTTTACAGCCTGCA
18S	ATGAGTCCACTTTAAATCCTTTAACGA	CTTTAATATACGCTATTGGAGCTGGAA	ATCCATTGGAGGGCAAGTCTGGTGC
HIF-1α	CCATGAGGAAATGAGAGAAATGC	GGCTTGTTAGGGTGCACTTCA	CACAGAAATGGCCCAGTGAGAAAAGGG
HO-1	CAGGGTGACAGAAGAGGCTAAGAC	TTGTGTTCCTCTGTCAGCATCAC	TCCTGCTCAACATTGAGCTGTTTGAGGA
IL-1β	TGAAAGACGGCACACCCA	GACAAACCGCTTTTCCATCTTC	CAGCTGGAGAGTGTGGATCCCAAACA
NLRP3	AGGACCCACAGTGTAACTTGC	CAGAGGTCAGAGCTGAACAACA	TAAGGCCGGAATTCACCAACCCCAGCT

### Cell culture and establishment of the hypoxia-reoxygenation condition

Kupffer cells and fatty hapetocyte were cultured in DMEM supplemented with 10% (v/v) fetal bovine serum, 1% penicillin/streptomycin (v/v). The cells were cultured at 37°C with the hypoxic condition (1% O_2_, 5% CO_2_, N_2_ as the base gas) in a modulator incubator chamber. Reoxygenation was obtained by replacing the cells in normal culture condition.

### *In vitro* experimental design

The Kupffer cells or fatty hepatocyte isolated from steatotic liver were randomly distributed to one of three groups as follows:

Group 1, control group: Kupffer cells or fatty hepatocyte;
Group 2, HR group: Kupffer cells or fatty hepatocyte suffered 4hrs of hypoxia and 6hrs of reoxygenation (HR);
Group 3, HR + ASTX group: Kupffer cells or fatty hepatocyte treated with ASTX before HR

### Effects of ASTX on inflammation and cytotoxic in Kupffer cells isolated from steatotic liver suffer HR

The method of Kupffer cells isolated from steatotic liver was established according to our previous work[18]. In details, liver was perfused with 15ml liver perfusion solution (3ml/min) and 20ml 0.1% collagenase solution was replaced at the same speed. Following digestion, the liver was transferred to a sterilized 100mm culture dish, grinded to homogenate. The liver homogenate was filtered through a 70μm cell strainer to remove undigested clots.

Cell suspension was centrifuged at 720g (BECKMAN COULTER Allegra X-12R, USA) for 7mins at 4°C. The top aqueous phase was discarded and the cell sediment was reserved. Cell sediments were re-suspended with 30ml DMEM and centrifuged at 720g for 7mins at 4°C, the aqueous phase was discarded, and the cell sediments was reserved. Cell sediments were re-suspended with 10ml GBSS-B and mixed with 14ml GBSS-A contain Nycodenz centrifuged (1900g, 20mins, 4°C). Harvested the cell of white layer re-suspended with 30ml GBSS-B centrifuged (50g, 7mins, 4°C). The top aqueous phase was transferred into a 50ml tube and centrifuged (720g, 7mins, 4°C). The cell sediments were seeded into 6-well plate by the density of 1×10^7^/well in culture medium and incubated in 5% CO_2_ incubator at 37°C. non-adherent cells were removed by washing with PBS after four hours, the adherent cells were Kupffer Cells.

Then the total RNA was extracted from Kupffer Cells, reverse transcribed to cDNA and detected by qRT-PCR.

The protein of Kupffer cells was disintegrated by radioimmunoprecipitation assay (RIPA) lysis buffer and stored in liquid nitrogen for western blot analysis.

### Effects of ASTX on anti-apoptosis in fatty hepatocyte isolated from steatotic liver suffer IRI

The method of liver perfusion and digestion is as same as the Kupffer cells’. The liver homogenate was re-suspended by culture medium, and passed through a 100μm filter for removing undigested clots.

Cell suspension was centrifuged at 80g (BECKMAN COULTER Allegra X-12R, USA) for 1min at 4°C. The top aqueous phase was discarded and the cell sediment was reserved. Cell sediments were re-suspended with 30% Percoll and centrifuged at 80g for 5mins at 4°C, the aqueous phase was discarded, and the cell sediments was reserved. Cell sediments were re-suspended with 45% Percoll and centrifuged at 80g for 2mins at 4°C. The top aqueous phase mixed with 2 fold of DMEM and transferred into a 50ml tube and centrifuged (80g, 1 min, 4°C). The cell sediments were seeded into 6-well plate by the density of 1×10^6^/well in DMEM medium with 10% FBS and 100U/ml Penicillin/Streptomycin and incubated in 5% CO_2_ incubator at 37°C. Twenty-four hours later non-adherent cells were removed by washing with PBS for 2 times, the adherent cells were fatty hepatocytes. Then the total RNA was extracted from fatty hepatocyte, reverse transcribed to cDNA and detected by qRT-PCR. The protein of Kupffer cells was disintegrated by radioimmunoprecipitation assay (RIPA) lysis buffer and stored in liquid nitrogen for western blot analysis.

### Reactive oxygen species (ROS) measured by immunofluorescent assay and flow cytometry

To verify the protective effect of the ASTX induced oxidative stress by hypoxia/reoxygenation. A ROS-sensitive fluorescent probe, H2DCFDA (CM-H2DCFDA; Invitrogen, Burlington, Ontario, Canada) was used to measure the content of ROS in Kupffer cells from steatotic liver suffering 4hrs hypoxia and 6hrs reoxygenation. The level of ROS was quantified by flow cytometry (GALLIOS, BECKMAN COULTER). In brief, 20,000 cells of every specimen were stained by CM-H2DCFDA checked by a flow cytometry for ROS measurement. Kupffer cells were incubated with 10μM CM-H2DCFDA, caputured by a fluorescence microscope (BX51, OLYMPUS, Osaka, Japan).

### Western blot analysis

Proteins from Kupffer cells and hepatocyte were homogenized with RIPA lysis buffer (Wako, Osaka, Japan) containing phosphatase inhibitor cocktail and protease inhibitor cocktail. Proteins were blotted onto PVDF membranes (Bio-Rad, Hercules, CA). The membranes were incubated with appropriate primary antibodies overnight at 4°C after blocked with skim milk for 1hr at room temperature. Afterwards, the membranes were washed three times with TBST and incubated with secondary antibodies for 1hr at room temperature. The blots were visualized by enhanced chemiluminescence. The primary antibodies were used are as follows: HO-1 (1:2000; Abcam, Cambridge, MA), Nrf-2 (1:2000; Santa Cruz Biotechnology), HIF-1α (1*2000; GeneTex), p-Akt (1:1000; Cell Signaling Technology), Akt (1:1000; Cell Signaling Technology), p-mTOR (1:1000; Cell Signaling Technology), mTOR (1:1000; Cell Signaling Technology), p-p38& p38 (1:2000; Cell Signaling Technology), p-ERK1/2& ERK1/2 (1:2000; Cell Signaling Technology), p-JNK (1:2,000; Cell Signaling Technology), JNK (1:4000; Cell Signaling Technology), α-tublin (1:4000; Santa Cruz Biotechnology) and GAPDH (1:4000; Santa Cruz Biotechnology).

### Statistical analysis

Data were presented as means ± SEM (means with standard errors) and analyzed statistically by a one-way ANOVA followed by Fisher’s protected least-significance difference test or the Mann–Whitney U test. A p<0.05 was considered to be statistically significant.

## Results

### ASTX pretreatment suppressed IRI in the steatotic liver

**Fig 1:**
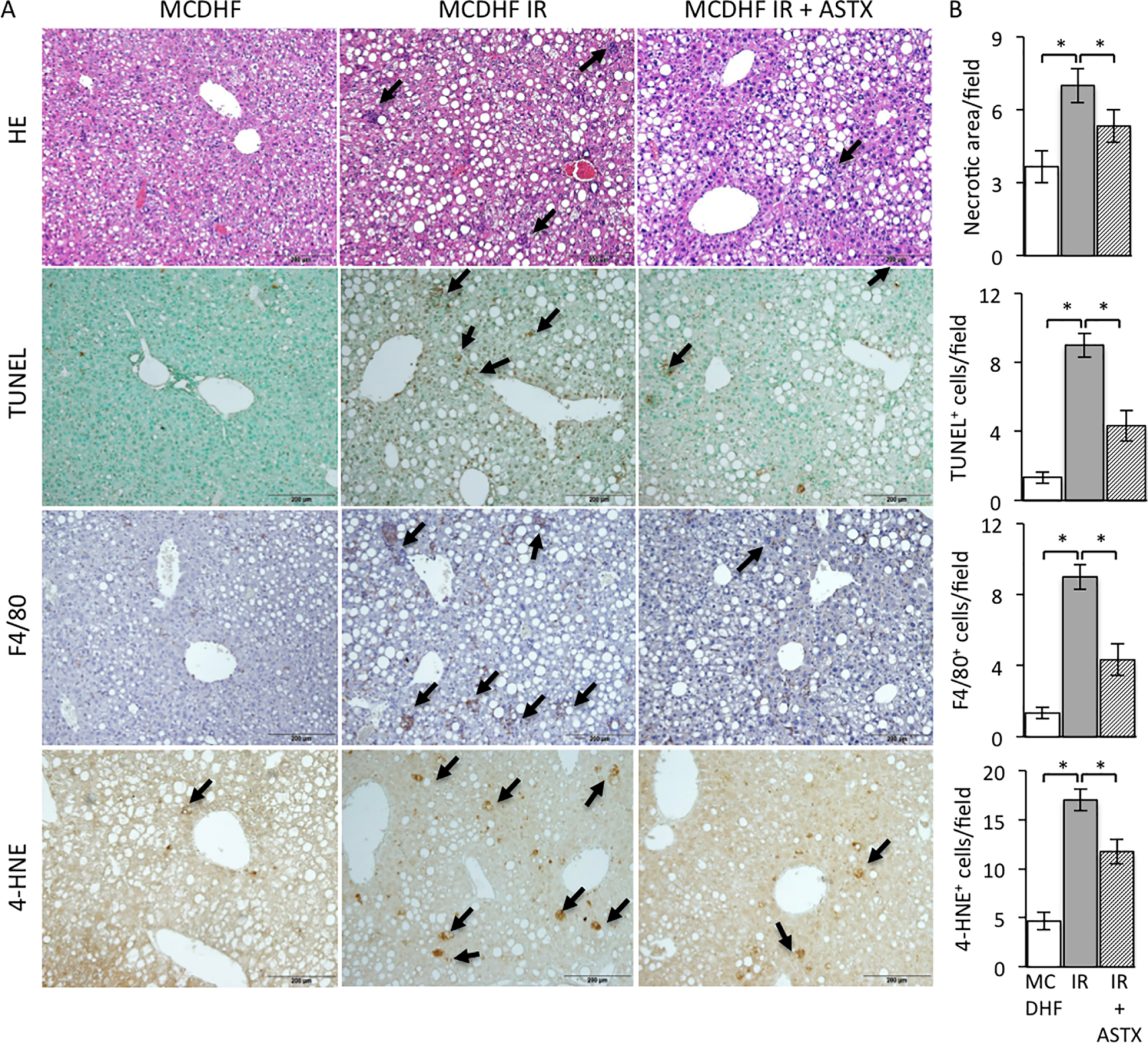
ASTX attenuated necrosis, infiltration of macrophages, oxidant stress and apoptosis as well as the biochemical parameters related to IR injury in the steatotic liver. (A) The necrotic cells, TUNEL-positive cells, F4/80-positive cells and 4-HNE-positive cells in MCDHF diet, IR and ASTX treatment group were indicated arrows. Scale bars represent 200μm, respectively. (B) The number of necrotic areas, TUNEL-positive cells, F4/80-positive cells and 4-HNE positive cells were counted. Data are expressed as the means ± SEM; *p < 0.05.

**Fig 2:**
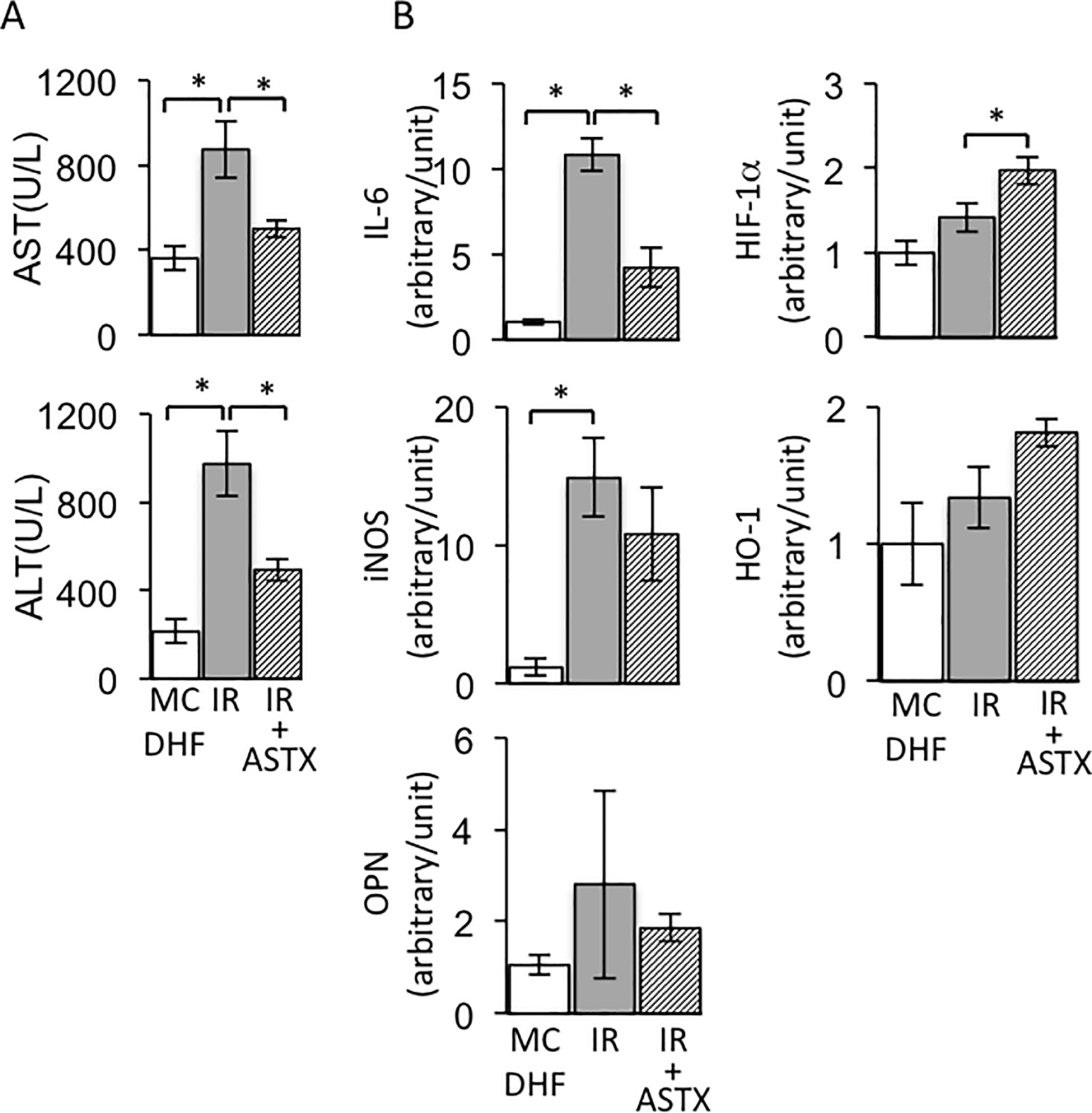
ASTX decreased the inflammatory cytokines expression and the biochemical parameters, but increased the HO-1 and HIF-1α expression in the steatotic liver under IR injury. (A) The serum ALT and AST levels of 15 minutes ischemia and 3 hours reperfusion. (B) The mRNA expression of IL-6, osteopontin (OPN), inducible nitric oxide synthase (iNOS), HO-1 and in HIF-1α in different groups. (Data are representative of 4~7 animals for each group). Data are expressed as the means ± SEM; *p < 0.05.

The necrotic area clearly showed that ASTX pretreatment decreased histological damage to the liver from IRI (Fig 1, first row). The amount of TUNEL-positive cells significantly increased compared with the simple steatotic liver, while ASTX administration dramatically suppressed the amount of TUNEL-positive cells (Fig 1, second row). Further, pre-treatment ASTX significantly depressed the distribution of F4/80-positive cells (Fig 1, third row). Furthermore, the amount of 4-HNE-positive cells significantly increased in the livers subjected to IR, and drastically decreased in the ASTX pre-treated steatotic livers subjected to IR (Fig 1, fourth row). In addition, the levels of AST and ALT were elevated, and were effectively reduced in the ASTX group compared with the IR group (Fig 2A).

### ASTX reduced mRNA expression of inflammatory cytokines and induced expressions of HO-1 and hypoxia-inducible factor (HIF)-1α in steatotic livers with IR

In contrast to the steatotic livers, high levels of these mRNA expressions were observed in the steatotic livers following IR (Fig 2B), whereas the ASTX-treatment group showed a significant reduction in expression of inflammatory cytokines. Further, as shown in Fig 2B, our result showed that the mRNA level of HIF-1α significantly and HO-1 slightly increased in the steatotic livers with ASTX pretreatment subjected to IR compared with sham steatotic livers and steatotic livers subjected to IR at the same time.

### ASTX decreased the content of ROS in Kupffer cells that were isolated from steatotic livers subjected to HR

**Fig 3:**
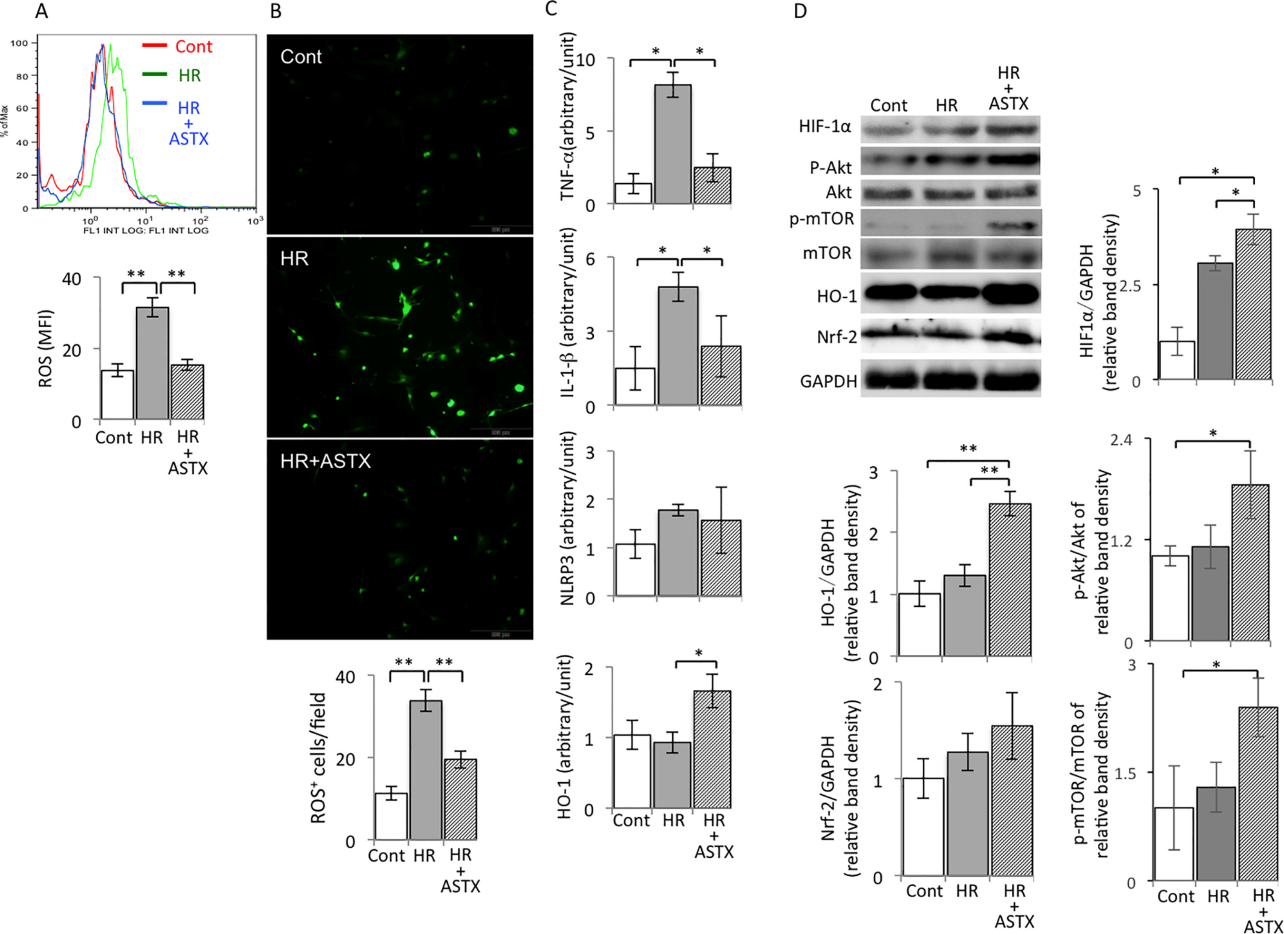
ROS, inflammatory cytokine mRNA expression, HO-1/Nrf-2 and Akt/mTOR/HIF1α pathways in ASTX treatment in Kupffer cells isolated from the steatotic liver subjected to HR. (A) The production of ROS in different groups of Kupffer cells determined by FACS(n = 3). (B) Fluorescence images of intracellular ROS production in Kupffer cells from steatotic liver mice subjected to HR loaded with ROS probe CM- H2DCFDA. Green fluorescence representing intracellular ROS was recorded using the confocol microscope(n = 3). (C) The mRNA expressions of inflammatory cytokines, as TNF-α, IL-1® and an inflammatory mediator, nlrp3 and the expression of HO-1in Kupffer cells suffering HR were checked by RT-PCR analysis(n = 3). (D) The protein expression of Kupffer cells subjected to HR with or without ASTX. The expression of HIF-1α, Akt, phosphorylated Akt, mTOR, HO-1and Nrf-2 were detected by western blotting. GAPDH was used as a loading control. (n = 3). Data are expressed as the means ± SEM; *p< 0.05, **p< 0.01. Data are representative of three independent experiments and indicate the mean ratio of triplicate results from each experiment.

The Kupffer cells subjected to HR produced significantly more ROS than control Kupffer cells. However, the content of ROS in Kupffer cells in steatotic livers subjected to HR significantly decreased in the group pretreated with ASTX (Fig 3A and 3B).

### ASTX regulated inflammatory cytokines, by enhanced HO-1, and Nrf2 expression and activated the Akt/mTOR/HIF1α pathway in Kupffer cells isolated from steatotic livers subjected to HR

Kupffer cells were isolated from steatotic livers pretreated with or without ASTX subjected to HR. The Kupffer cells from ASTX pretreatment decreased the mRNA expression of inflammatory cytokines such as IL-1β, TNF-α, and showed a tendency to reduce the NOD-like receptor 3 (Nlrp3) inflammasome component compared with Kupffer cells from steatotic livers subjected to HR. In contrast, the mRNA expression of HO-1 in Kupffer cells from steatotic livers subjected to HR was significantly up-regulated after ASTX pretreatment. In addition, the expression of transcription factor Nrf-2, which may play a role in inducing HO-1 transcription, also increased when pretreated with ASTX. In addition, to further investigate the HO-1/Nrf-2 signaling pathway on Kupffer cells, we observed that the protein expressions of HO-1 and Nrf-2 were up-regulated after ASTX treatment in Kupffer cells from steatotic livers subjected to HR (Fig 3C and 3D). Expression of HIF-1α, and phosphorylation of mTOR and Akt were increased in ASTX-treated Kupffer cells from steatotic livers suffered to HR (Fig 3D).

### ASTX alleviated apoptosis by corrected Bax/Bcl-2 ratio and depressed the phosphorylated level of p38, ERK, and JNK in steatotic hepatocytes isolated from steatotic livers subjected to HR

**Fig 4:**
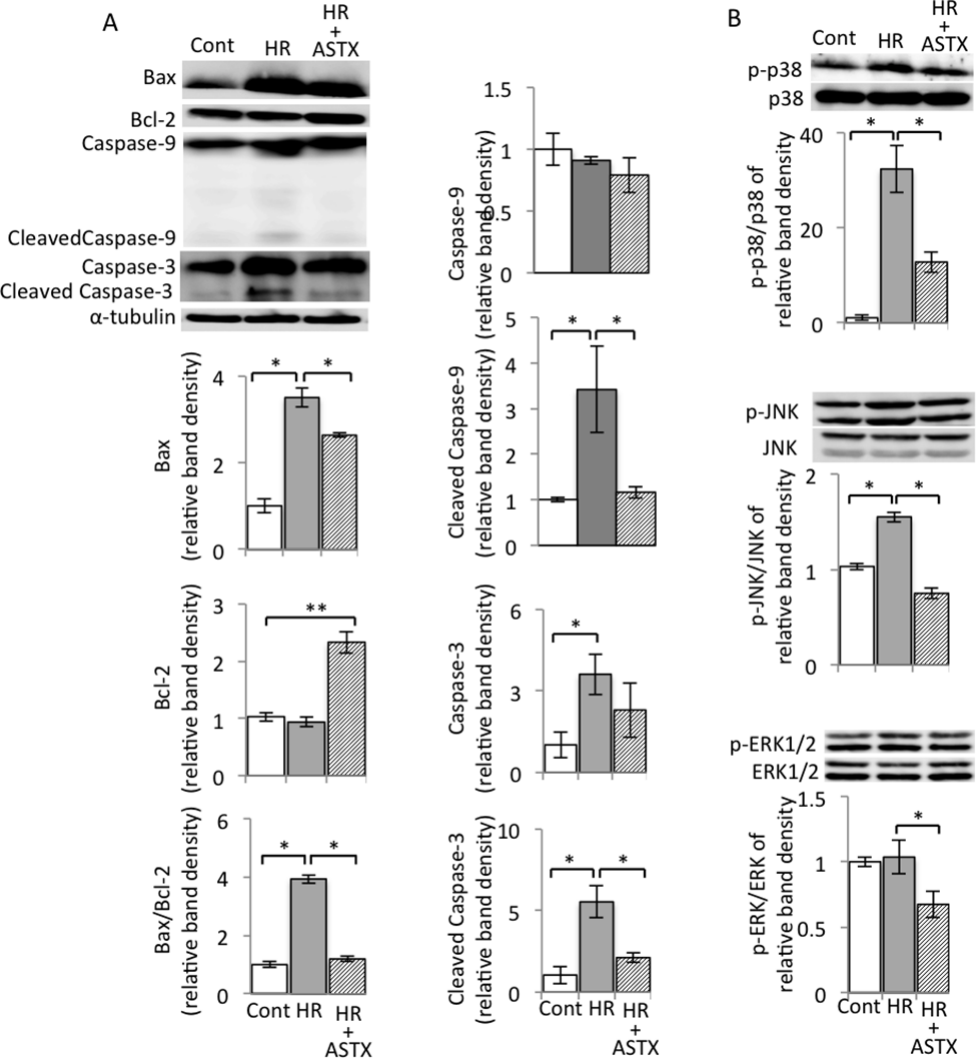
ASTX prevented steatotic primary hepatocyte apoptosis when hepatocytes subjected to HR. (A) Expressions of Bax, Bcl-2, cleaved caspase-9, caspase-9, cleaved caspase-3, caspase-3 of steatotic primary hepatocyte by hypoxia/reoxygenation injury were detected by western blot analysis. Representative blot (left) and quantified protein levels (right) are shown(n = 3). (B) Protein expression of p38 MAPK, p-p38 MAPK, ERK, p-ERK, JNK and p-JNK was detected by western blots(n = 3). α-tubulin was used as a loading control. Representative blot (up) and quantified protein levels (down) are shown. Data are expressed as the means ± SEM; *p< 0.05, **p< 0.01. Data are representative of three independent experiments and indicate the mean ratio of triplicate results from each experiment.

As shown in Fig 4A, HR up-regulated the expression of Bax while slightly down-regulating or having almost the same level of Bcl-2 as the control group, while treatment with ASTX dramatically aggravated the changes of Bax and Bcl-2 expression. The Bax/Bcl-2 ratio increased in steatotic primary hepatocytes subjected to HR, but significantly decreased after ASTX treatment. Furthermore, there were 6 and 5.5-fold increases in the level of cleaved caspase-9 and -3 in steatotic primary hepatocytes subjected to HR, respectively, compared to the control group. In contrast, the protein level of cleaved caspase-9 and -3 markedly decreased in the ASTX group. Interestingly, HR markedly increased the expression of caspase-3 compared with control group. ASTX also decreased the caspase-3 protein expression induced by HR, while the inhibitory effect of ASTX on cleaved caspase-3 protein expression was more obviously than on caspase-3.

The proteins of the MAPK family including subgroups p38 MAPK, ERK and JNK can be activated through phosphorylation in response to apoptosis and extracellular stimulation. After HR, the oxidative stress reaction in steatotic hepatocytes isolated from steatotic livers is essential for apoptosis. In our study, the phosphorylation levels of p38 MAPK, ERK, JNK were measured. The analysis results from the proportion of proteins reflected that the phosphorylation level of p38 MAPK and JNK in steatotic hepatocytes significantly increased after HR and showed the tendency of high levels of phosphorylated ERK, which was consistent with apoptosis. After oral administration of ASTX, a decline in p-p38 MAPK, p-ERK and p-JNK expression in steatotic hepatocytes was observed. These results indicated that ASTX inhibited the phosphorylation of the critical proteins of the MAPK family that are responsible for mitigating cell apoptosis (Fig 4B).

## Discussion

In this study, we aimed to assess the effects of ASTX in a mouse steatotic liver IRI model by assessing histology, liver enzymes, mRNA expression of inflammatory cytokines, ROS production, apoptosis-related molecule expression and phosphorylation of MAPK signaling molecules *in vivo* and *in vitro*.

This is the first attempt to use ASTX for the treatment of IRI in the steatotic liver. Improvements were observed in the ASTX-treated group, including: 1) the number of cell deaths; 2) increased number of activated macrophages; 3) deposition of oxidative products; 4) AST and ALT leakage; 5) inflammatory cytokine expression including IL-6, inducible nitric oxide synthase and osteopontin, and 6) expression of pro-survival molecules, HIF-1α and HO-1. The clinical outcome of our trial demonstrated that ASTX is a novel therapeutic agent for attenuating IRI in a steatotic mouse liver. Further, we estimated the impact of ASTX on HR for isolated Kupffer cells and hepatocytes from steatotic mouse livers by appraising ROS production, mRNA and/or protein expression of inflammatory molecules, including TNF-α, IL-1β, NLRP3, HO-1 and Nrf-2, and protein expression of apoptosis-related molecules, including Bax, Bcl-2, caspase-3 and 9 and phosphorylation of MAPK signaling molecules, including p38, JNK and ERK.

Due to its complexity, the mechanism of hepatic IRI has not yet been elucidated, but growing evidence points towards the accumulation of ROS and the expression of inflammatory factors as critical factors leading to liver damage [19]. Further, IRI of the steatotic liver is more serious than the normal liver [3]. In order to avoid the influence of estrogen-mediated protective pathway in female mice that attenuates hepatocellular IR injury, we chose the eight weeks old male C57BL/6 mice to establish the steatotic liver and suffer IR [20, 21]. There are some kinds of diet to induce fatty liver mouse model, like high fat diet and MCD diet. If we use high fat diet, hepatic steatosis, oxidative stress, and inflammation will be observed at 12–16 weeks [22]. But in MCD diet mouse model, 3 weeks was enough. The one of the purposes of our article was establishing a model of fatty liver and suffering IR injury to mimic IR injury in human fatty liver as a donor in liver transplantation. In the view of time and effect and our aim, we chose MCD diet to establish fatty liver mouse model.

ASTX produced by marine life is a natural antioxidant and can improve the outcome of liver grafting. Some reports have shown that ASTX dissolved in olive oil had no negative effects and also had protective effects against oxidative stress [23]. Recently, Ni et al reported that ASTX was more useful in preventing and treating the steatotic liver compared with vitamin E in mice [24]. Further, several reports demonstrated that ASTX attenuates hepatic [25, 26], retinal [27], and renal [28] IRI in the rodent model. Therefore, it is necessary to investigate the effects of ASTX on ROS, inflammation and apoptosis induced by hepatic IRI in the steatotic liver.

The rapid rise of AST, ALT and lipid droplets in hepatocytes is a significant manifestation of the successful establishment of hepatic IR in the steatotic liver mouse model. HE staining showed the presence of inflammatory infiltration and necrosis, which is consistent with pathologic manifestation. ASTX inhibited the serum level of liver enzymes and inflammatory infiltration and necrosis of parenchymal cells (Figs 1 and 2).

Kupffer cells are the resident macrophages located in hepatic sinusoids, so they are the first batch of cells to make contact with exogenous immune-reactive material, which then stimulate the cells. It has been shown that a high-fat diet increases the amount of Kupffer cells and is associated with their pro-inflammatory status [29]. In the early phase of hepatic IR, released damage-associated molecular patterns (DAMP) caused by ischemia injury bind to the Toll-like receptor (TLR) on Kupffer cells, leading to Kupffer cell activation [30]. Activated Kupffer cells respond by releasing a large amount of inflammatory cytokines including IL-6, TNF-α, IL-12, IL-1β, chemokines and endogenous ROS to further enhance the inflammatory reaction [31, 32]. ASTX can be used as a guarantee for inhibiting oxidative damage through various mechanisms, including radical scavenging, lipid peroxidation inhibition and regulation of oxidative-stress-related gene expression [33, 34]. Pretreatment with ASTX could decrease ROS generation and lipid peroxidation in rat models of ischemic stroke [35]. In the hepatocarcinogenesis model, the protective effects of ASTX attributed to the activation of the Nrf-2/ARE pathway ameliorated DNA damage and induced oxidative stress by cyclophosphamide [36]. In our study, ASTX enhanced HO-1 expression slightly, but inhibition of oxidative damage in the steatotic liver was indicated by immunostaining of 4-HNE, a marker of oxidative stress (Figs 1 and 2). Further, we conducted comprehensive examinations to check the beneficial effects of ASTX in the *in vitro* model of HR. We found that administration of ASTX facilitated the activation of Nrf-2 leading to the up-regulation of the Nrf-2-regulated enzyme HO-1 in Kupffer cells, which isolated from the steatotic liver to resist inflammatory cytokines expression induced by HR (Fig 3D and S1 Fig). Simultaneously, ASTX reduced the ROS generation in Kupffer cells subjected to HR (Fig 3A and 3B). Extracellular oxygen sensing is mainly mediated by the HIF pathway. The Akt/mTOR pathway stabilizes and upregulates HIF-1α[36]. We observed that HIF-1α, phosphorylation of mTOR and Akt expression were significantly increased in ASTX-treated Kupffer cells from steatotic livers suffered to HR, indicating that ASTX also preconditioning exerts its protective potentiation effects on Kupffers through the HIF pathway (Fig 3D and S1 Fig). The expression of the cyto-protective protein HO-1 was more remarkable in isolated Kupffer cells, and reached up to 93% purity [18] compared to liver homogenate (Figs 2B and 3D).

Apoptosis as well as necrosis plays an important role in hepatic IRI [37]. In our study, a great number of TUNEL-positive cells evidently demonstrated deterioration in IR-induced apoptosis as compared to the MCDHF group (Fig 1). In contrast, a marked decrease was observed in the amount of TUNEL-positive cells and the levels of AST and ALT in serum as a result of pretreatment with ASTX (Figs 1 and 2A). These results suggest that ASTX could effectively protect parenchymal cells. The pathways of ASTX in hepatocyte protection by anti-apoptosis effect have not yet been clarified. Steatotic livers are less tolerant to acute injury induced by hepatic ischemia and reperfusion, and hypoxia/reoxygenation-induced apoptosis is also more severe in steatotic hepatocytes than normal hepatocytes. In our study, primary hepatocytes from the steatotic liver were used to investigate the anti-apoptosis role of ASTX. The change in O_2_ concentrations from 1% to 21% corresponds to the processes of HR *in vitro* and remarkably mimics the processes of IR *in vivo*, and could therefore increase the apoptosis of steatotic primary hepatocytes. It has been demonstrated that HR enhances apoptotic activity in human hepatocytes by increasing the death receptor 5 [38]. Further, numerous key factors in HR-mediated hepatocyte apoptosis have been demonstrated, such as proapoptotic Bax and antiapoptotic Bcl-2, and cleaved caspase-3 and 9. In our study, HR triggered apoptotic activity in steatotic hepatocytes by increasing the Bax/Bcl-2 ratio and the increased expressions of cleaved caspases-3 and -9, indicating that caspase-regulated cell apoptosis is crucial in IR-induced liver injury [25]. In addition, an interaction mechanism exists between Bcl-2 and caspase-3, in which Bcl-2 plays a role of negative regulation in the expression of caspase-3 [39]. In the present study, pre-treatment with ASTX significantly ameliorated the HR-induced upgrade of Bax, downgrade of Bcl-2, upregulation of caspase-3/9 and cleavage of caspase-3/9 in the steatotic hepatocytes subjected to HR. Cleaved caspase-3 is an activated form of caspase-3. To form an active enzyme, caspase need cleavage adjacent to aspartates to the liberation of a large and a small subunit forming α_2_β_2_ tetramer[40]. The incidence has been reported the expression of caspase family, not only cleavage of caspase-3 and 9 [41, 42], but also expression of caspase-3 and 9 [43, 44] can be activated by ischemia/reperfusion. We showed that the expression of caspase-3 and cleavage of caspase-3 were enhanced in steatotic primary hepatocyte suffered HR. ASTX markedly decreased cleaved caspase-3 expression but slight decreased the expression of caspase-3, suggesting that ASTX protected the primary hepatocyte by more specifically reducing the expression of cleaved caspase-3 than inhibiting the expression of caspase-3. These data suggested that ASTX inhibits I/R-induced hepatic parenchymal cells apoptosis with down-regulation of caspase-3/9 (Fig 4A and S2A Fig).

Cell death was also related to MAPK signaling, including p38 MAPK, JNK and ERK [45, 46]. Many studies have demonstrated that p38 MAPK could be phosphorylated by the release of ROS-regulated Bax translocation [47, 48]. JNK has also been reported as the mediator of apoptosis in IR [49]. Furthermore, HR could increase the level of caspases and pro-apoptotic cofactors through ERK up-regulation [50]. Recent reports have indicated that p-ERK1/2, p-JNK, and p-p38 were significantly activated by IR, and pretreatment of ASTX decreased the up-regulation of p-ERK1/2, p-p38 and p-JNK [25, 49]. In agreement with this study, our data showed that HR increased p-p38 and p-JNK, but decreased p-ERK expression in hepatocytes from the steatotic liver. In contrast, pretreatment with ASTX inhibited increased expression of p-p38, p-JNK and decreased expression of p-ERK lower than the control (Fig 4B and S2B Fig). Our results suggest in line with previous reports that the protective effect of ASTX against HR-induced apoptosis in steatotic hepatocytes relied on the inhibition of ERK, JNK and p38 signaling pathways. We have performed Oil Red O staining to evaluate the effects of ASTX on liver steatosis. However, it seems that the steatosis and hepatic triglycerides of three groups remained unchanged or the difference did not reach statistical significance (S3 Fig). These results suggested that the effects of ASTX on IRI in steatotic liver is not due to the improvement of steatosis.

In conclusion, our results demonstrated the protective effect of ASTX in the steatotic liver affected by IRI. The histopathological features of IRI, including necrosis, apoptosis infiltration of macrophages, lipid peroxidation products, liver enzyme leakage and inflammatory cytokine expression were corrected by ASTX. Inhibition of ROS production and inflammatory cytokine expression, induction of HO-1/Nrf-2 in Kupffer cells, and inactivation of MAPK may be involved in the inhibitory mechanism of ASTX against IRI in the steatotic liver. The decline of cleaved caspase-3/9, down-regulation of Bax expression and up-regulation of Bcl-2 level in hepatocytes may also play a role.

## Conclusions

This is the first attempt to use ASTX for the treatment of IRI in the steatotic liver. ASTX attenuates the injury level in the steatotic liver ischemia reperfusion injury including decreased ROS production and inflammatory cytokine expression, while increased expression of HIF-1α, HO-1 and Nrf-2 and activated the Akt/mTOR/HIF-1α in Kupffer cells, decreased the expression of cleaved caspase-3/9, Bax, while increased Bcl-2 level in hepatocyte. Pretreatment with ASTX has a protective effect and is a safe therapeutic treatment for IRI, including for liver transplantation of the steatotic liver.

## Supporting information

S1 Fig ASTX enhanced HO-1 and Nrf-2 expression in Kupffer cells. Immunoblots of HO-1 and Nrf-2 levels in Kupffer cells treated with 10μM astaxanthin subjected to HR (n = 3).
(TIF)

S2 Fig Effect of ASTX on steatotic hepatocytes apoptosis and MAPK signal pathway. (A) Immunoblots of Bax, Bcl-2, cleaved caspase-9, caspase-9, cleaved caspase-3, caspase-3 levels in steatotic hepatocytes subjected to HR (n = 3). (B) Immunoblots of p38 MAPK, p-p38 MAPK, ERK, p-ERK, JNK, p-JNK levels in steatotic hepatocytes subjected to HR (n = 3).
(TIF)

S3 Fig Effect of ASTX on liver steatosis and hepatic triglycerides. (A) Oil Red O staining in liver from MCDHF diet, IR and ASTX treatment group. Representative images were shown at original magnification (x200), and scale bars = 100μm. (B) Oil Red O staining positive cells were counted and hepatic triglycerides were measured (n = 3).
(TIF)
