# Supplementary figures and images for: Astaxanthin prevents ischemia-reperfusion injury of the steatotic liver in mice
Source: PLoS One. 2017 Nov 9;12(11):e0187810. doi: 10.1371/journal.pone.0187810 (PMC5679630; doi:10.1371/journal.pone.0187810)

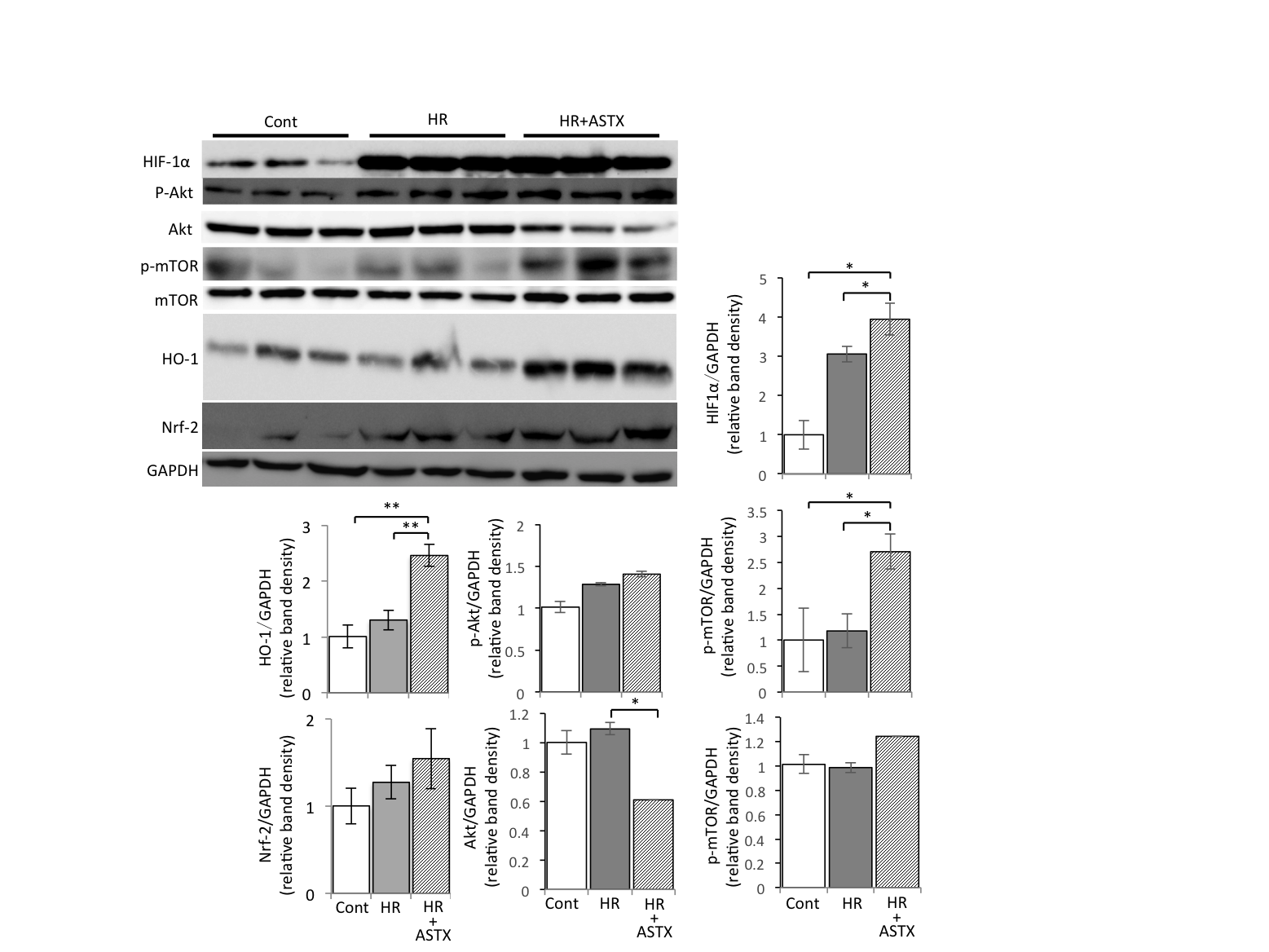

Supplement: S1 Fig — Immunoblots of HO-1 and Nrf-2 levels in Kupffer cells treated with 10μM astaxanthin subjected to HR (n = 3). (TIF) [file pone.0187810.s001.tif]

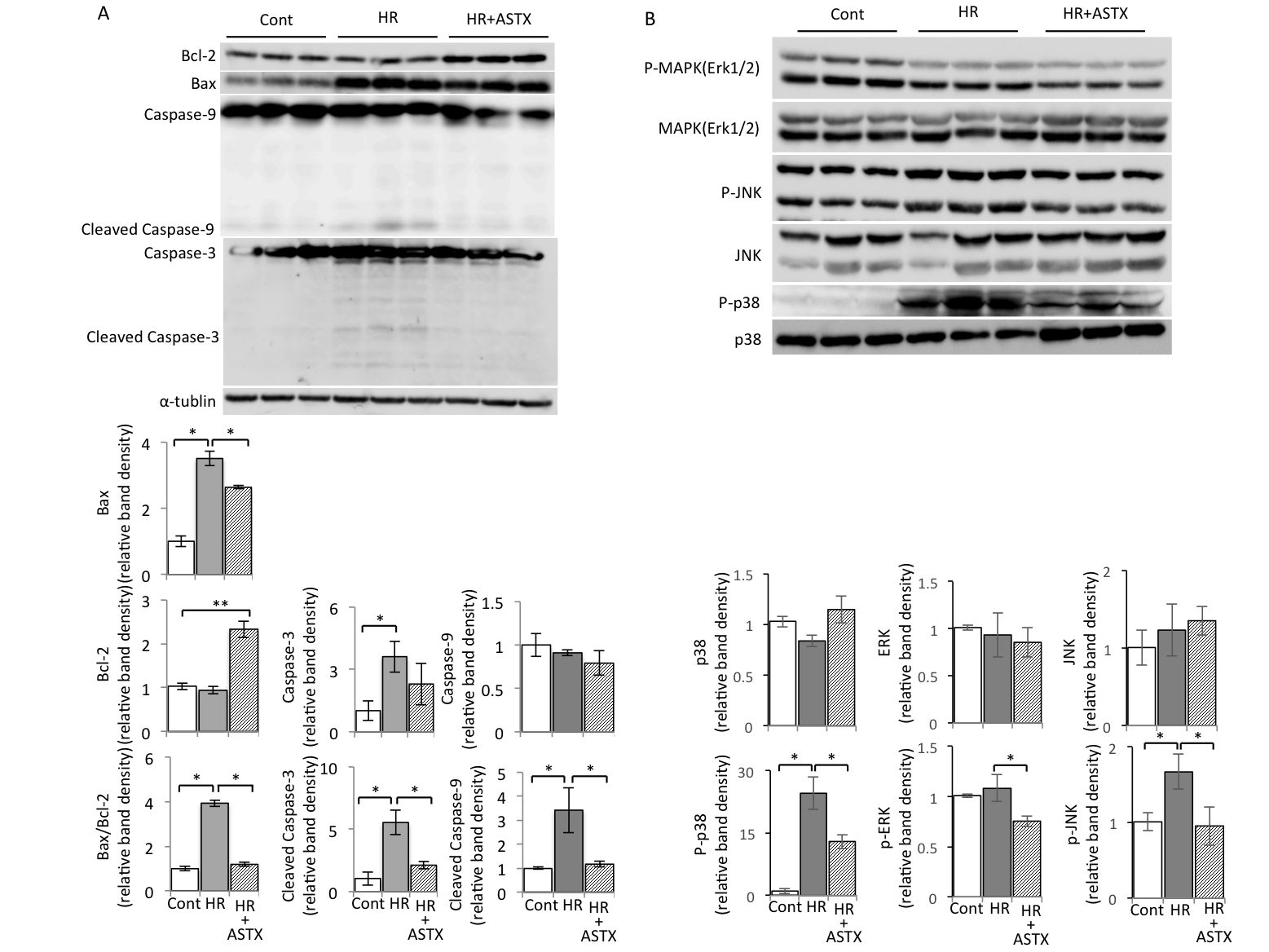

Supplement: S2 Fig — (A) Immunoblots of Bax, Bcl-2, cleaved caspase-9, caspase-9, cleaved caspase-3, caspase-3 levels in steatotic hepatocytes subjected to HR (n = 3). (B) Immunoblots of p38 MAPK, p-p38 MAPK, ERK, p-ERK, JNK, p-JNK levels in steatotic hepatocytes subjected to HR (n = 3). (TIF) [file pone.0187810.s002.tif]

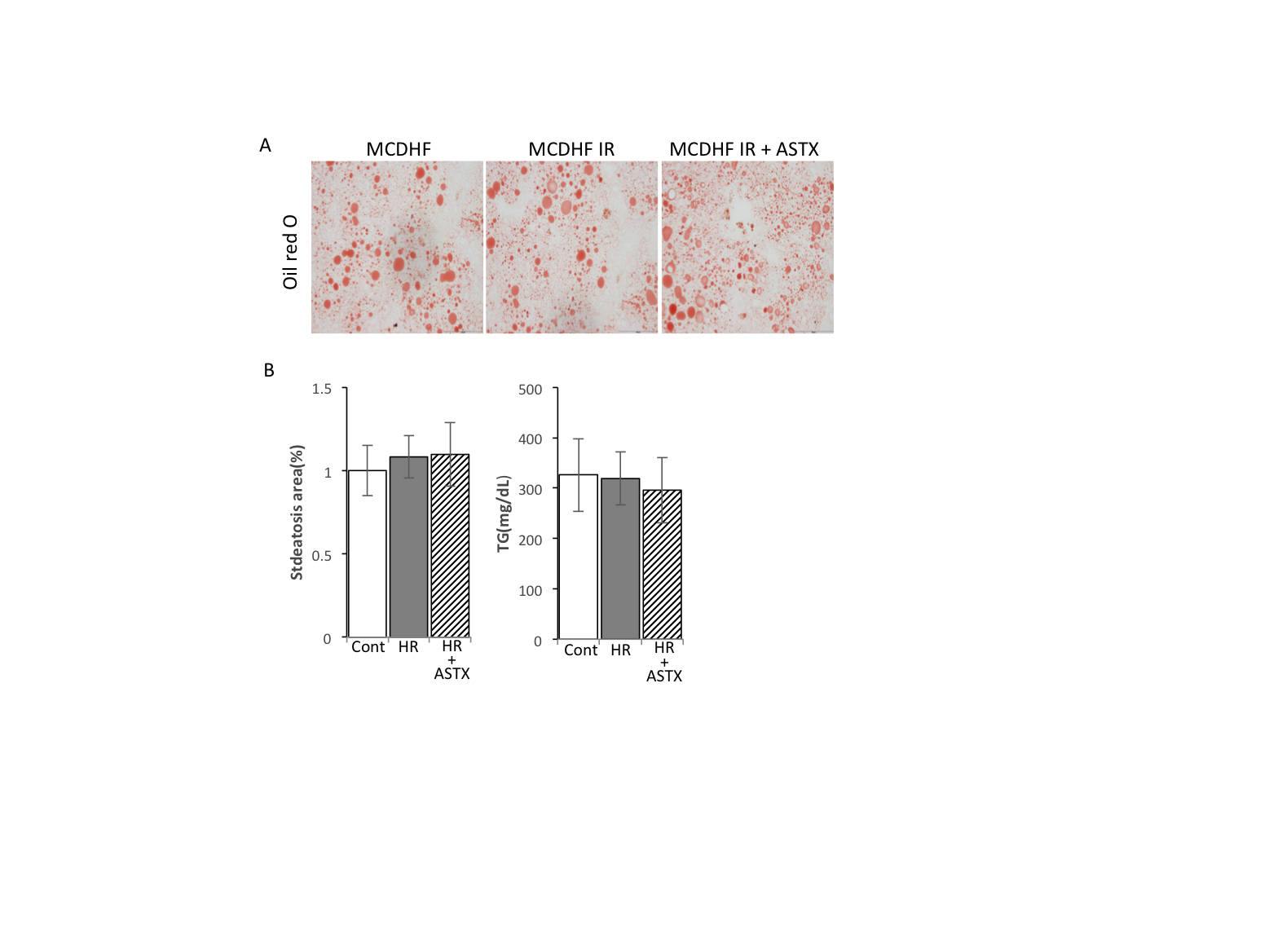

Supplement: S3 Fig — (A) Oil Red O staining in liver from MCDHF diet, IR and ASTX treatment group. Representative images were shown at original magnification (x200), and scale bars = 100μm. (B) Oil Red O staining positive cells were counted and hepatic triglycerides were measured (n = 3). (TIF) [file pone.0187810.s003.tif]
